# Risk Factors and Outcomes of Extensively Drug-Resistant Gram-Negative Bacilli in Neonates with Late-Onset Sepsis

**DOI:** 10.3390/antibiotics15020166

**Published:** 2026-02-04

**Authors:** Sanchat Sanchainara, Anucha Thatrimontrichai, Praew Chareesri, Pattima Pakhathirathien, Manapat Praditaukrit, Gunlawadee Maneenil, Supaporn Dissaneevate

**Affiliations:** Division of Neonatology, Department of Pediatrics, Faculty of Medicine, Prince of Songkla University, Songkhla 90110, Thailand; ssanchat@medicine.psu.ac.th (S.S.); praew.c@psu.ac.th (P.C.); ppattima@medicine.psu.ac.th (P.P.); manapat.p@psu.ac.th (M.P.); mgunlawa@medicine.psu.ac.th (G.M.); dsupapor@medicine.psu.ac.th (S.D.)

**Keywords:** *Acinetobacter baumannii*, bacteremia, bacterial meningitis, bacterial multidrug resistance, extensive antibacterial drug resistance, *Klebsiella pneumoniae*, neonatal late-onset sepsis, neonatal sepsis, newborn, septic shock

## Abstract

**Background/Objective**: To identify the risks and outcomes of extensively drug-resistant Gram-negative bacilli (XDR-GNB) in neonates. **Methods**: This retrospective case–control study (1995–2024) included neonates with late-onset sepsis (*n* = 132) and XDR-GNB bacteremia (*n* = 26) compared with those without XDR-GNB (*n* = 106). **Results**: Median gestational age was 31 weeks and birth weight 1540 g. The prevalence of XDR-GNB was 19.7%. The most common XDR-GNB and non-XDR-GNB pathogens were *Acinetobacter baumannii* and *Klebsiella pneumoniae*. Sepsis onset occurred earlier in the XDR-GNB group than in the non-XDR-GNB group (7.0 vs. 12.5 days, *p* = 0.005). In multivariable analysis using Firth’s penalized likelihood method, the XDR-GNB group was more likely to have gastrointestinal anomalies (adjusted odds ratio 3.81, 95% confidence interval 1.24–12.01, *p* = 0.02) and history of umbilical arterial catheterization (adjusted odds ratio 3.04, 95% confidence interval 1.21–7.95, *p* = 0.02) compared to the non-XDR-GNB group. The XDR-GNB group had higher rates of septic shock (50.0% vs. 18.9%, *p* = 0.002) and inadequate empiric antimicrobial therapy (34.6% vs. 13.2%, *p* = 0.02). The non-susceptibility rates to third-generation cephalosporins, gentamicin, carbapenems, amikacin, and colistin were 83.3%, 58.3%, 48.1%, 30.4%, and 4.4%, respectively. **Conclusions**: Empirical colistin treatment is warranted for neonates in high-XDR environments who exhibit septic shock and have specific risk factors, such as gastrointestinal anomalies or the presence of an umbilical arterial catheter. Multimodal interventions, including antimicrobial stewardship programs, have been used to prevent or reduce the incidence of neonatal XDR-GNB sepsis.

## 1. Introduction

Neonatal sepsis is a life-threatening disease with a high morbidity risk that affects critically ill newborns [1], resulting in a global case-fatality rate (CFR) of 2–50% [2,3,4,5,6]. Early onset sepsis (EOS) and late-onset sepsis (LOS) were defined as positive blood cultures before or 72 h after birth with clinical sepsis, respectively [7]. Advances in perinatal and neonatal management have helped reduce this rate over the last 30 years (1990–2019, 36.6 to 17.5 per 1000 live births) [8]. In addition, between 2000 and 2023, the mortality rate associated with neonatal sepsis decreased from 5.2 to 3.7 per 100,000 live births (−30.3% reduction) [9]. Concurrently, the mean number of LOS episodes per admission decreased by 4.0% per year [10]. This downward trend has been overshadowed by the escalating burden of antimicrobial resistance, which is largely driven by the excessive use of antibiotics. Although some modern antibiotics have a broader spectrum of action than those used in the past, antibiotic overuse persists, resulting in antimicrobial resistance (AMR) [11].

In Australia [10] and Mexico [12], LOS pathogens have demonstrated higher levels of resistance than EOS pathogens. Gram-negative bacillus (GNB) sepsis is associated with a higher incidence of septic shock than Gram-positive sepsis [13]. The incidence of late-onset GNB bacteremia, especially due to the *Enterobacteriaceae* family, has increased in neonatal intensive care units (NICU) [14]. Moreover, GNB-related sepsis is associated with higher CFR in premature neonates [15]. Finally, neonatal sepsis contributes to prolonged hospital stay, increased healthcare costs, and adverse neurodevelopmental outcomes [16,17].

Modern neonatal care protocols have increased survival rates of preterm and surgical neonates. Adequate empirical antimicrobial treatment (EAT) for neonatal sepsis may help reduce CFR. In contrast, neonates may die if they receive inadequate EAT (IEAT) in highly multidrug-resistant (MDR) cases. Prolonged antibiotic use in very low birthweight infants in the first week of life is associated with subsequent LOS, necrotizing enterocolitis, or death [18]. However, antimicrobial-resistant pathogens may colonize due to frequent and prolonged broad-spectrum antimicrobial use [19]. The emergence of MDR-GNB sepsis is of particular concern because of the off-label use of carbapenems and colistin during the neonatal period, with implications for global health [20,21].

From 2010 to 2021, antibiotic consumption decreased in the Western Pacific and European WHO regions, but increased in Southeast Asia, Africa, and Latin America [22]. In Australia, the proportion of colonizing GNB isolates resistant to third-generation cephalosporins or gentamicin has increased by 2.9% per year [10]. The prevalence of MDR-GNB colonization showed an upward trend [23]. The incidence of MDR-GNB neonatal sepsis in Taiwan, Hungary, Thailand, and Egypt was 19%, 34%, 66%, and 83%, respectively [24,25,26,27]. The CFRs for MDR-GNB sepsis in Hungary, Taiwan, Thailand, and Egypt were 24%, 29%, 29%, and 79%, respectively [21,22,23,24].

Although the rates of AMR and access to drugs vary globally, exposure to MDR has increased, driving poor outcomes, and creating challenges in the NICUs [28,29,30]. However, studies on the risk factors, types of organisms, and outcomes associated with extensively drug-resistant (XDR) GNB and LOS in neonates are limited. Herein, we aimed to perform a case–control study to evaluate potential risk factors and outcomes in neonates with XDR-GNBs during LOS.

## 2. Results

During 1995–2024, 88,858 and 12,961 neonates were born and admitted to the NICU, respectively. A total of 363 neonates developed GNB sepsis. Neonates who were outborn (*n* = 173), had EOS (*n* = 43), or had non-fermenting GNB sepsis (*n* = 15; *Burkholderia cepacia* [*n* = 3], *Serratia marcescens* [*n* = 6], *Serratia rubidaea* [*n* = 1], *Salmonella* [*n* = 1], and an unidentified genus and species [*n* = 4]) were excluded. The late-onset GNB sepsis rate in inborn neonates was 1.65 per 1000 live births (147 per 88,858 neonates). The rate of late-onset GNB sepsis in the admitted neonates (including both inborn and outborn neonates) was 2.47% (320/12,961).

**Figure 1:**
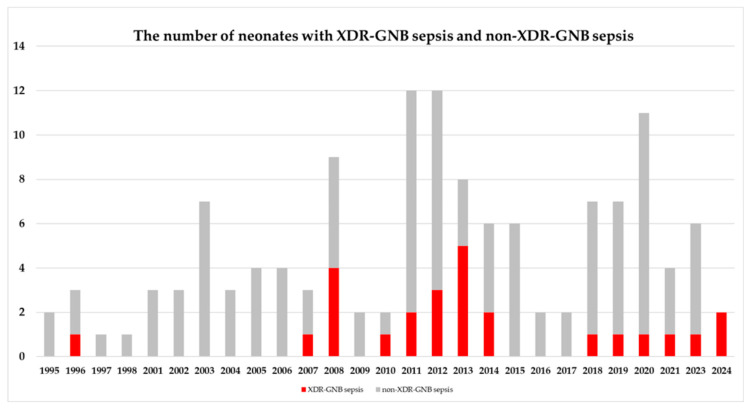
Number of neonates with extensively drug-resistant Gram-negative bacilli (XDR-GNB) and non-XDR-GNB in late-onset sepsis in each year.

In total, 132 neonates were included in the analysis. The median (interquartile range) gestational age (GA) and birth weight (BW) were 31 (28–36) weeks and 1540 (897–2581) grams, respectively. The prevalence of XDR-GNB was 19.7% (26/132 patients). The baseline characteristics of the XDR-GNB and non-XDR-GNB groups are presented in Table 1. None of these factors differed. In 2013, the XDR-GNB sepsis showed the highest prevalence (62.5%, 5/8) in Figure 1.

The onset of sepsis in the XDR-GNB group was earlier than that in the non-XDR-GNB group (median [IQR] 7.0 [4.3–16.2] days vs. 12.5 [7.3–23.5] days, *p* = 0.005). In addition, the XDR-GNB group experiences umbilical arterial catheterization (UAC) at a higher rate than the non-XDR-GNB group (53.8% vs. 28.3%, *p* = 0.03) (Table 2).

The most common GNB pathogens were *Klebsiella pneumoniae* (*n* = 60), *Acinetobacter baumannii* (*n* = 40), and *Enterobacter cloacae* (*n* = 14) (Table 3). The most common pathogen causing XDR-GNB sepsis was *A. baumannii* (80.8%, 21/26). Time to positivity in XDR-GNB sepsis was longer than that in non-XDR-GNB sepsis (median [IQR] 3.7 [2.8–4.5] days vs. 3.1 [2.4–3.9] days, *p* = 0.04). The IEAT in the XDR-GNB group (34.6%) was higher than that in the non-XDR-GNB group (13.2%). In XDR-GNB sepsis, the susceptibility to colistin and aminoglycosides was 90.0% and 23.1%, respectively. In non-XDR-GNB sepsis, the susceptibility to colistin, ciprofloxacin, and carbapenems was 97.1%, 73.3%, and 63.2%, respectively.

From all pathogens (Table 4), non-susceptibility was defined as either an intermediate or resistant result, based on the final available antibiogram and interpreted according to current clinical breakpoints. The percentages of non-susceptibility to third-generation cephalosporins, gentamicin, carbapenems, amikacin, and colistin were 83.3%, 58.3%, 48.1%, 30.4%, and 4.4%, respectively. Only Enterobacteriaceae family, the percentages of non-susceptibility to third-generation cephalosporins, gentamicin, carbapenems, amikacin, and colistin were 84.0%, 55.7%, 27.5%, 27.1%, and 5.9%, respectively.

Risk factors associated with *p*-values of <0.2 in univariable analysis were entered into multivariable analysis (Table 5). In the final model, the XDR-GNB group was more likely to have gastrointestinal (GI) anomalies (adjusted odds ratio [OR] 1.21, 95% confidence interval [CI] 1.02–1.43, *p* = 0.03) and history of UAC (adjusted OR 1.21, 95% CI 1.06–1.40, *p* = 0.007) than non-XDR-GNB group.

The XDR-GNB group was more likely to develop septic shock than the non-XDR-GNB group (50.0% vs. 18.9%, respectively; Table 6). However, there were no significant differences in mortality, length of stay, or daily hospital costs. The CFRs caused by *Pseudomonas aeruginosa*, *Acinetobacter* spp., *K. pneumoniae*, *E. cloacae*, and *Escherichia coli* were 37.5% (3/8), 27.9% (12/43), 21.7% (13/60), 21.4% (3/14), and 14.3% (1/7), respectively.

## 3. Discussion

In this 30-year retrospective case–control study, XDR-GNB accounted for 19.7% of late-onset GNB sepsis in neonates. Compared with non-XDR-GNB sepsis, XDR-GNB sepsis occurred significantly earlier, was independently associated with GI anomalies and prior UAC, and was more frequently complicated by septic shock and IEAT. *A. baumannii* and *K. pneumoniae* were the predominant pathogens, and high non-susceptibility rates were observed for third-generation cephalosporins, aminoglycosides, and carbapenems, while colistin retained activity against most isolates. These findings highlight the clinical severity and therapeutic challenges of XDR-GNB sepsis and provide a framework for comparison with existing regional and international data.

The emergence of XDR-GNB sepsis indicates that most standard EAT are no longer effective, making prevention and control particularly challenging in NICU settings. In this study, neonates with GI anomalies or a history of UAC were at an increased risk of XDR-GNB sepsis, underscoring the importance of device- and procedure-related exposures. In NICUs with a high prevalence of *A. baumannii* colonization, neonates presenting with septic shock may require empirical treatment with carbapenems combined with colistin because of the high susceptibility observed in this pathogen [31,32,33,34]. Conversely, in units with low *A. baumannii* colonization pressure, carbapenems-based EAT alone may be adequate for initial management. Importantly, a broader-spectrum EAT should be promptly de-escalated once culture results are available, highlighting the critical role of local XDR epidemiology in guiding empirical antibiotic selection at the unit or country level.

Sepsis onset occurred earlier in the XDR-GNB group than in the non-XDR-GNB group, suggesting acquisition from the NICU environment rather than stepwise resistance development. Antimicrobial stewardship programs have been shown to reduce antibiotic consumption and mitigate resistance driven by colonization pressure. The NICU environment may act as a reservoir of AMR transmission, particularly *A. baumannii,* which commonly colonizes the respiratory tract and is a well-recognized cause of ventilator-associated pneumonia (VAP). In settings with high XDR-*A. baumannii* prevalence, implementation of environmental cleaning bundles—including ventilatory circuit care— is essential to reduce transmission. Multimodal prevention and control strategies remain central to limiting the spread of resistant bacteria in the NICU [1].

The prevalence of late-onset GNB sepsis among inborn neonates in this study (1995–2024) was 1.65 per 1000 live births, which is comparable to reported in Sweden (0.24 per 1000 live births, 2006–2016 [35]), Australia and New Zealand (1.14 per 1000 live births, 1992–2002, 20 neonatal units [36]) and Taiwan (1.36 per 1000 live births, 2004–2011 [37]). The prevalence of late-onset GNB sepsis in both inborn and outborn neonates in this study (2.47%) was lower than that reported in Turkey (4.4%, 2008 [38]), India (4.3%, 2017–2019 [39]; 4.8%, 2019–2021 [40]), and Taiwan (5.8%, 2004–2011 [37]).

Most previous studies have focused on the prevalence of MDR-GNB sepsis in neonates [12,28,30,35,37,41] with limited data specifically addressing XDR-GNB sepsis. In the present study, XDR-GNB accounted for 19.7% (26/132) of late-onset GNB sepsis cases, a proportion comparable to that reported in a Mexican study (23.7%, 9/38, from August 2021 to April 2023) [12]. However, the common XDR pathogens in the Mexican study were different from those identified in this study (*P. aeruginosa* [23.5%, 4/17], *K. pneumoniae* [27.3%, 3/11], and *E. coli* [22.2%, 2/9]).

The distributions of pathogens varied across geographical regions. The most common pathogens identified in this study were *K. pneumoniae* and *Acinetobacter* spp. (75%). The most common pathogen in late-onset GNB sepsis was *K. pneumoniae* similar to that reported in a meta-analysis [42], and studies in China (25 NICUs [43] and 5 neonatal units [44]), Taiwan [37], India [39,40], South and Southeast Asia (10 neonatal units in five countries [45]), and Italy [46]; however, *E. coli* was the most common organism in the UK (30 neonatal units [47]), Canada (10 NICUs [48]), Sweden (10 neonatal units [35]), and Germany [49]. *Acinetobacter* spp. are common AMR pathogens in neonates in South and Southeast Asia [45]. High non-susceptibility rates for *K. pneumoniae* and *Acinetobacter* spp. in China, and South and Southeast Asia were similar to those observed in this study, except in Italy and Canada, where higher susceptibility rates were reported) (Table 7).

The onset of XDR sepsis was earlier than that of non-XDR sepsis (7.0 versus 12.5 days). We speculate that neonates with XDR sepsis are more likely to acquire drug-resistant pathogens from NICU colonization or ventilatory circuits directly or indirectly into the bloodstream than they are to develop resistance by themselves. Moreover, previous antibiotic exposure did not differ between the groups. Previous studies have provided limited data on the onset of XDR sepsis in neonates. In a recent study from India, the onset of healthcare-associated bloodstream infections in NICUs occurred during days 3–7 (54.2%) and 8–14 (30.0%) after admission [39].

Neonates with XDR-GNB sepsis were more likely to have a history of GI anomalies and UAC than those with non-XDR-GNB sepsis. In neonates with GI anomalies, *Enterobacteriaceae* family, as well as *Klebsiella* spp., *E. coli*, and *E. cloacae* may directly migrate from the gut microbiome and be replaced by MDR or XDR strains via postsurgical conditions or prolonged gastric tube. Furthermore, *Acinetobacter* spp. and *P. aeruginosa* usually colonize the ventilator circuit. Intubated neonates require frequent monitoring of arterial blood gases from the UAC. Device utilization (endotracheal intubation and umbilical venous or central venous catheterization) rate was higher in the XDR sepsis group than in the non-XDR sepsis group; however, the difference was not statistically significant. Moreover, environmental reservoir-hospital surfaces, sewage, and stream water-harboring resistant biofilm producers reinforce their role in the persistence and dissemination of XDR- *Klebsiella* spp. [50]. Therefore, environmental disinfection protocols require strict adherence, especially in the operating room and NICU, to reduce colonization pressure from XDR pathogens.

Peak prevalence of XDR-GNB sepsis was observed in 2013. In 2014, protocols featuring enhanced environmental cleaning and the introduction of heat and moisture exchangers were implemented. These measures led to a sustained decrease in endotracheal carbapenem-resistant *A. baumannii* colonization and concomitantly reduced the need for broad-spectrum antimicrobial agents (both carbapenem and colistin) in the NICU from 2015 to 2017 [51].

We found that the IEAT was higher in patients with XDR sepsis than in those with non-XDR sepsis (34.6% vs. 13.2%). Neonates with XDR sepsis (50.0%) were more likely than those with non-XDR sepsis (18.9%) to develop septic shock. However, the associated mortality rate and healthcare costs did not differ. Neonatal infection by *P. aeruginosa* was associated with the highest mortality in late-onset GNB sepsis (37.5% [3/8]), similar to findings in Australia and New Zealand (52.3% [46/88], 1992–2002, 20 neonatal units [36]) and Taiwan (68.8% [11/16], 2004–2011) [37]. The CFRs from other late-onset GNB sepsis cases in this and previous studies [36] were 14.3–27.9% and 13.7–23.8%, respectively.

The primary strength of this study lies in providing robust empirical evidence, which is a significant advantage given the paucity of data on neonatal XDR sepsis. Our findings captured the prevalence, risk factors, causative pathogens, susceptibility patterns, and outcomes associated with late-onset XDR-GNB sepsis. Clinically, the selection of EAT must align with local epidemiology to achieve adequate coverage against resistant pathogens. In high-XDR settings, the prompt initiation of broad-spectrum antibiotic treatment for neonates with septic shock, followed by streamlining based on antimicrobial susceptibility results, is essential. This antimicrobial stewardship approach can help improve clinical outcomes, reducing mortality rates and treatment costs associated with XDR sepsis.

This study has some limitations. First, it is based on long-term surveillance data, and changes in the neonatal care protocols were observed during the study period, resulting in heterogeneity that may have introduced both bias and confounding effects. Second, the retrospective nature of this study introduced limitations due to missing data, particularly regarding antimicrobial susceptibility. Furthermore, data concerning specific risk factors for XDR sepsis, such as the duration of ventilator and central line use, were often omitted from medical records. Third, our analysis focused on risk factors and outcomes associated with XDR sepsis in neonates. Notably, these findings reflect the prevalence (each neonate) of sepsis rather than its incidence (each episode). Risk factor data were collected solely upon initial positive hemoculture, and the outcomes represented the patient status before hospital discharge. Fourth, a geographic bias limits the generalizability of our results. Fifth, despite the long study period, the number of cases and controls was small. Therefore, future research should focus on multicenter studies or larger cohorts to obtain a larger sample size, which would allow for a more comprehensive collection of AMR data and a deeper understanding of neonatal XDR sepsis. Ultimately, as an observational study, any clinical implications are restricted to informing routine EAT strategies within high XDR environments.

## 4. Materials and Methods

### 4.1. Study Setting, Design, and Patient Domains

This study was conducted in the NICU of the Songklanagarind Hospital, a teaching hospital affiliated with the Prince of Songkla University in Thailand. The hospital records approximately 2500–3500 live births annually, with a level IV NICU admitting 400–550 inborn and outborn neonates per year. The study protocol was approved by the Human Research Ethics Committee of the Prince of Songkla University (approval number: 67–550–1–1), with a waiver of informed consent.

This was a retrospective case–control study. We identified neonates with Gram-negative sepsis using records from the hospital’s clinical microbiological laboratory and the Songklanagarind database. The patient domains comprised all neonates admitted to the Songklanagarind Hospital NICU between 1 January 1995, and 31 December 2024.

The inclusion criteria were all neonates with culture-proven GNB sepsis, including all species of *Enterobacteriaceae* and certain non-*Enterobacteriaceae* species (only either *P. aeruginosa* or *Acinetobacter* spp.) identified from positive blood cultures. Neonates who were outborn, had EOS or non-fermenting GNB sepsis (with the exception of *P. aeruginosa* and *Acinetobacter* spp.), or had unidentified GNB organisms were excluded. After identifying patient domains through the microbiological laboratory database, hospital records were reviewed only for the first episode of culture-proven sepsis. The risk factors for both XDR-GNB and non-XDR-GNB cases were extracted and evaluated from birth until the onset of sepsis. The percentage of non-susceptibility to each pathogenic organism was analyzed only from the first episode of sepsis.

### 4.2. General and Specific Neonatal Care

An empirical antibiotic regimen for EOS typically involves a combination of ampicillin and gentamicin. In contrast, treatment for LOS utilizes broad-spectrum agents, such as cefotaxime or ceftazidime combined with amikacin, cefoperazone/sulbactam, carbapenems, or colistin. Escalation to a broader spectrum of antibiotics is indicated if the infant’s clinical condition fails to improve within 48–72 h or based on the antibiogram report, subject to the clinical judgment of the attending neonatologist or pediatrician. EAT was immediately de-escalated to a narrow spectrum based on the reported antibiogram or discontinued entirely upon confirmation of a negative culture result, and clinical improvement was observed.

The EAT available for severe sepsis or septic shock have evolved over time; imipenem, ciprofloxacin, meropenem, cefoperazone/sulbactam, and colistin were introduced into clinical practice in 1992, 2000, 2005, and 2007, respectively. The recommended total duration for antibiotic prescriptions varies based on the specific condition: 3–5 days for clinical sepsis, 7–14 days for VAP, 10–14 days for Gram-positive bacteremia, 14 days for GNB bacteremia, and 21 days for bacterial meningitis. Routine screening for group B *Streptococcus* infections is not standard practice in Thailand.

From 1995 to 2013, routine neonatal care included: (1) reused ventilator circuits, with heated humidifiers and a heated wire in the inspiratory limb only and a water trap in the expiratory limb, cleaned by pasteurization; and (2) 0.5% sodium hypochlorite to clean the NICU environment (walls and rails) and the vacuum-suction base. Since 2014, multimodal interventions have included: (1) use of heat and moisture exchangers with dual heated wires and the permeable microcell technique; (2) use of 0.05% sodium hypochlorite for the neonatal environment (inside and outside the incubator and the radiant warmer), followed by cleaning, dry linens, and 0.5% sodium hypochlorite to clean the NICU environment [51]; and (3) elective high-frequency oscillatory ventilation initiated as the primary mode after intubation. New noninvasive ventilation methods include nasal high-frequency oscillatory ventilation and nasal (synchronized) intermittent positive pressure ventilation used for post-extubation support [52], and (4) oral care with maternal milk or sterile water [53]. The study period was divided into three 10-year intervals: 1995–2004, 2005–2014, and 2015–2024.

### 4.3. Neonatal Sepsis

EOS and LOS were defined as positive blood cultures before or 72 h after birth with clinical sepsis, respectively [7]. In the absence of universally accepted neonatal Sepsis-3 criteria, sepsis in this study was defined as culture-proven infection accompanied by suggestive clinical features, a definition consistent with previous neonatal literature. Blood specimen was obtained at least 1 mL [7] under sterile conditions and processed in an automatic blood culture machine (BACTEC FX™ (Becton Dickinson, Sparks, MD, USA) or BacT/Alert™ (bioMérieux, Marcy-l’Étoile, France)). Susceptibility testing of each patient was performed using the disk diffusion method (zone diameter interpretive criteria) according to the latest updated editions of the Clinical and Laboratory Standards Institute guidelines. The antimicrobial susceptibility profiles were classified as susceptible, intermediate, or resistant. Non-susceptibility was defined as intermediate or resistant according to the final antibiogram.

An XDR organism was defined as non-susceptibility to at least one agent in all but two or fewer antimicrobial categories (i.e., bacterial isolates remained susceptible to only one or two categories). These classes include aminoglycosides, carbapenems, fluoroquinolones, non-extended-spectrum cephalosporins, extended-spectrum cephalosporins, cephamycins, folate pathway inhibitors, glycylcyclines, monobactams, penicillins, penicillin/beta-lactamase inhibitors, phenicols, phosphonic acids, polymyxins, and tetracyclines [54].

### 4.4. Definitions

Small, appropriate, and large for GA were defined as BW below the 10th, 10th–90th, and above the 90th percentile, respectively. Central nervous system anomalies were defined as spinal bifida or surgical neurological sequelae. Congenital heart anomalies were defined as congenital complicated heart disease, cyanotic heart disease, or acyanotic heart disease with signs of heart failure. Gastrointestinal anomalies were defined as esophageal atresia, pyloric stenosis, small or large bowel obstruction, omphalocele, or gastroschisis.

The diagnosis of VAP was made based on the National Healthcare Safety Network guidelines for infants aged less than 1 year [55]. Previous antibiotic exposure was defined as intravenous antibiotic use for at least 72 h before the culture was obtained. The aminoglycosides include amikacin and gentamicin. Cephalosporins include cefotaxime and ceftazidime. Carbapenems include imipenem and meropenem. IEAT was defined as the administration of antibiotics for more than 48 h post-hemoculture, which either failed to cover the identified causative microorganisms or was ineffective against resistant pathogens.

Septic shock was defined as clinical sepsis plus GA–dependent hypotension, tachycardia, or reduced tissue perfusion requiring vasopressor agents to maintain blood pressure within 48 h after the onset of sepsis [56]. The CFR was defined as the crude mortality rate observed after the onset of sepsis. The length of stay was defined as the duration of admission until the patient was discharged from the hospital. Daily hospital cost was calculated as the total hospital cost divided by the length of hospital stay (1 U.S. dollar [USD] = 30 baht).

### 4.5. Statistical Analysis

The R program (version 4.5.1; R Foundation for Statistical Computing, Vienna, Austria [57]), including the EpiCalc package (version 4.1.0.1; Epidemiology Unit, Prince of Songkla University, Songkhla, Thailand [58]) and the logistf package (version 1.26.1), was used for data analysis. Categorical variables are presented as frequency and percentage and were compared using the χ^2^ test or Fisher’s exact test. Nonparametric continuous variables are presented as medians (IQR) and were compared using the Mann–Whitney U test (Ranksum test).

Univariable and multivariable analyses were performed. Variables with *p*-values of <0.2 in the univariable analysis, along with those of biological plausibility, were included in a multivariable backward stepwise logistic regression model using Firth’s penalized likelihood method. Adjusted OR and 95% CI were computed for variables that were independently associated with XDR and non-XDR GNB sepsis. Outcomes associated with XDR-GNB sepsis were also analyzed. All *p*-values were 2-tailed, and *p* < 0.05 was considered statistically significant.

## 5. Conclusions

The pervasive threat of XDR infections remains a global health concern. In the LOS cohort, GI anomalies and UAC were identified as factors associated with an increased likelihood of XDR-GNB sepsis, relative to the non-XDR-GNB group. XDR-GNB sepsis was associated with a greater frequency of septic shock than was observed in the non-XDR-GNB cohort. Critically, sepsis onset occurred earlier in the XDR-GNB group than in the non-XDR-GNB group. Infection prevention and control protocols, thorough environmental cleaning, and robust antimicrobial stewardship programs require strict implementation in areas with high MDR resistance. In high-XDR settings, broader empirical antimicrobial coverage may be considered for neonates presenting with septic shock and relevant risk factors, with prompt de-escalation based on microbiological results and clinical response. Finally, further research is warranted to elucidate the risk factors in broader neonatal and pediatric XDR infection populations and to facilitate more personalized antimicrobial therapy for LOS [11,19].

## Figures and Tables

**Table 1:** Characteristics of neonates with extensively drug-resistant Gram-negative bacilli (XDR-GNB) and non-XDR-GNB in late-onset sepsis.

Factors on Date of Birth	XDR-GNB Sepsis (*n* = 26)	Non-XDR-GNB Sepsis (*n* = 106)	*p*-Value
GA, weeks *	31.5 (28–34)	31.0 (28–37)	0.49 ^1^
BW, g *	1765 (1103–2158)	1450 (893–2634)	0.88 ^1^
BW compared to GA, *n* (%)			0.49 ^2^
Appropriate for GA	23 (88.5)	81 (76.4)	
Small for GA	3 (11.5)	22 (20.8)	
Large for GA	0 (0)	3 (2.8)	
Male, *n* (%)	16 (61.5)	64 (60.4)	>0.99 ^3^
Cesarean section, *n* (%)	20 (76.9)	71 (67.0)	0.46 ^3^
5 min Apgar score *	8 (7–9)	8 (7–9)	0.34 ^1^
Endotracheal intubation at birth, *n* (%)	21 (80.8)	66 (62.3)	0.12 ^3^
Surfactant administration, *n* (%)	6 (23.1)	25 (23.6)	>0.99 ^3^
Congenital anomalies, *n* (%)			
Central nervous system anomalies	3 (11.5)	14 (13.2)	>0.99 ^2^
Congenital heart anomalies	11 (42.3)	46 (43.4)	>0.99 ^3^
Gastrointestinal anomalies	8 (30.8)	16 (15.1)	0.09 ^2^
Study period			0.02 ^3^
1995–2004	1 (3.8)	22 (20.8)	
2005–2014	18 (69.2)	44 (41.5)	
2015–2024	7 (26.9)	40 (37.7)	

* Data are presented as median (interquartile range). ^1^ Ranksum test ^2^ Fisher’s exact test ^3^ χ^2^ test. BW, birth weight; GA, gestational age.

**Table 2:** Factors associated with late-onset sepsis in neonates with extensively drug-resistant Gram-negative bacilli (XDR-GNB) and non-XDR-GNB.

Factors on Date of Sepsis	XDR-GNB Sepsis (*n* = 26)	Non-XDR-GNB Sepsis (*n* = 106)	*p*-Value
History of ventilator-associated pneumonia, *n* (%)	4 (15.4)	10 (9.4)	0.48
History of total parenteral nutrition use, *n* (%)	21 (80.8)	72 (67.9)	0.30
Umbilical arterial catheterization, *n* (%)	14 (53.8)	30 (28.3)	0.03
Umbilical venous catheterization, *n* (%)	13 (50.0)	34 (32.1)	0.14
Central venous catheterization, *n* (%)	5 (19.2)	15 (14.2)	0.55
Previous antibiotic exposure, *n* (%)	24 (92.3)	90 (84.9)	0.52
Aminoglycosides	21 (80.8)	78 (73.6)	0.61
Cefotaxime or Ceftazidime	12 (46.2)	54 (50.9)	0.83
Cefoperazone plus sulbactam	5 (19.2)	17 (16.0)	0.77
Carbapenems	8 (30.8)	23 (21.7)	0.47

**Table 3:** Pathogenic organisms in neonates with extensively drug-resistant Gram-negative bacilli (XDR-GNB) sepsis and non-XDR-GNB sepsis.

Pathogens	XDR-GNB Sepsis (*n* = 26)	Non-XDR-GNB Sepsis (*n* = 106)	*p*-Value
Time to positivity, d *	3.7 (2.8–4.5)	3.1 (2.4–3.9)	0.04
List of organisms			<0.001
*Acinetobacter baumannii*	21 (80.8)	19 (17.9)	
*Acinetobacter junii*	0 (0)	2 (1.9)	
*Acinetobacter lwoffii*	0 (0)	1 (0.9)	
*Enterobacter cloacae*	0 (0)	14 (13.2)	
*Escherichia coli*	0 (0)	7 (6.6)	
*Klebsiella pneumoniae*	4 (15.4)	56 (52.8)	
*Pseudomonas aeruginosa*	1 (3.8)	7 (6.6)	
Non-susceptibility, *n* (%)			
Aminoglycosides	20 (76.9)	69 (65.1)	0.36
Carbapenems **	24/25 (96.0)	39 (36.8)	<0.001
Cephalosporins	26 (100.0)	84 (79.2)	0.007
Ciprofloxacin	23 (92.0)	28 (26.7)	<0.001
Penicillin **	26 (100.0)	88/100 (88.0)	0.07
Colistin **	2/20 (10.0)	2/70 (2.9)	0.21
Inadequate empiric antimicrobial therapy, *n* (%)	9 (34.6)	14 (13.2)	0.02

* Data are presented as median (interquartile range). ** Indicated in complete data.

**Table 4:** Non-susceptibility to each pathogenic organism.

Pathogens	Aminoglycosides, *n* (%)		Carbapenems, *n* (%)	Cephalosporins, *n* (%)	Colistin, *n* (%)
	Amikacin	Gentamicin			
*Acinetobacter* spp.	11/38 (28.9)	23/38 (60.5)	37/43 (86.0)	40/43 (93.0)	0/34 (0)
*Enterobacter cloacae*	6/14 (42.9)	10/14 (71.4)	4/14 (28.6)	12/14 (85.7)	1/9 (11.1)
*Escherichia coli*	0/6 (0)	2/6 (33.3)	3/7 (42.9)	4/7 (57.1)	0/4 (0)
*Klebsiella pneumoniae*	13/50 (26.0)	27/50 (54.0)	15/59 (25.4)	52/60 (86.7)	2/38 (5.3)
*Pseudomonas aeruginosa*	5/7 (71.4)	5/7 (71.4)	4/8 (50.0)	2/8 (25.0)	1/5 (20.0)
Total	35/115 (30.4)	67/115 (58.3)	63/131 (48.1)	110/132 (83.3)	4/90 (4.4)

Some data was unavailable.

**Table 5:** Univariable and multivariable analyses of the risk factors for extensively drug-resistant Gram-negative bacilli sepsis in neonates.

Risk Factors	Univariable Analysis		Multivariable Analysis	
	Odds Ratio (95% Confidence Interval)	*p*-Value	Adjusted Odds Ratio (95% Confidence Interval)	*p*-Value
Study period (reference: 1995–2004)	1			
2005–2014	1.28 (1.06–1.54)	0.01	4.27 (0.93–41.02)	0.06
2015–2024	1.11 (0.91–1.35)	0.29	1.34 (0.23–14.20)	0.76
Gastrointestinal anomalies	1.18 (0.99–1.41)	0.06	3.81 (1.24–12.01)	0.02
Endotracheal intubation since birth	1.14 (0.99–1.31)	0.08		
History of umbilical arterial catheterization	1.20 (1.04–1.38)	0.01	3.04 (1.21–7.95)	0.02
History of umbilical venous catheterization	1.13 (0.98–1.30)	0.09		

**Table 6:** Outcomes of neonates with extensively drug-resistant Gram-negative bacilli (XDR-GNB) sepsis and non-XDR-GNB sepsis.

Outcomes	XDR-GNB Sepsis (*n* = 26)	Non-XDR-GNB Sepsis (*n* = 106)	*p*-Value
Septic shock within 48 h of sepsis, *n* (%)	13 (50.0)	20 (18.9)	0.002
Case-fatality rate, *n* (%)	10 (38.5)	22 (20.8)	0.10
Length of stay, d *	49 (22–75)	54 (27–95)	0.40
Daily hospital cost, USD *	USD 220.1 (130.5–423.9)	USD 264.9 (136.0–577.9)	0.48

* Data are presented as median (interquartile range).

**Table 7:** Comparison of non-susceptibility to *Klebsiella pneumoniae* and *Acinetobacter* spp. in this study and previous studies.

Pathogens	Previous and These Studies (year), [Reference]	Percentage of All Organisms	Aminoglycosides, *n* (%)		Carbapenems, *n* (%)	Cephalosporins, *n* (%)
			Amikacin	Gentamicin		
*K. pneumoniae*	This study (1995–2024)	45.5% ^1^	13/50 (26.0)	27/50 (54.0)	15/59 (25.4)	40/43 (93.0)
	China (2017–2019), [43]	49.0% ^1^	N/A	41/196 (20.9)	26/196 (13.3)	124/196 (63.3)
	Italy (2011–2022), [46]	34.4% ^1^	0/32 (0)	1/33 (3.0)	0/32 (0)	3/33 (9.1)
	South and Southeast Asia (2019–2020), [45]	44.9% ^2^	(22.7)	(52.9)	(17.1)	(86.7)
	Canada (2015–2021) [48]	20.4% ^2^	0/196 (0)	11/196 (5.7)	0/196 (0)	35/196 (17.8)
*Acinetobacter* spp.	This study (1995–2024)	30.3% ^1^	11/38 (28.9)	23/38 (60.5)	37/43 (86.0)	52/60 (86.7)
	China (2017–2019), [43]	7.0% ^1^	N/A	4/28 (14.3)	8/28 (28.6)	11/28 (39.3)
	Italy (2011–2022), [46]	2.1% ^1^	0/2 (0)	0/2 (0)	0/2 (0)	0/2 (0)
	South and Southeast Asia (2019–2020), [45]	28.7% ^2^	(29.7)	(87.5)	(76.5)	N/A
	Canada (2015–2021) [48]	4.0% ^2^	0/38 (0)	0/38 (0)	0/38 (0)	N/A

^1^ Only late-onset sepsis and ^2^ combination of early-onset and late-onset sepsis caused by *Acinetobacter* spp., *Enterobacter cloacae*, *Escherichia coli*, *Klebsiella* spp., and *Pseudomonas aeruginosa.* N/A, not available.

## Data Availability

Raw data supporting the conclusions of this study are provided by the authors upon request. These data are not publicly available because of privacy concerns.

## Abbreviations

The following abbreviations are used in this manuscript:

AMR	antimicrobial resistance
CFR	case-fatality rate
EAT	empirical antimicrobial therapy
GI	gastrointestinal
GNB	Gram-negative bacilli
IEAT	inadequate empirical antimicrobial therapy
LOS	late-onset sepsis
MDR	multidrug-resistant
NICU	neonatal intensive care unit
UAC	umbilical arterial catheterization
VAP	ventilator-associated pneumonia
XDR	extensively drug-resistant
